# Long-Term Stability of *Lavandula x intermedia* Essential Oil Nanoemulsions: Can the Addition of the Ripening Inhibitor Impact the Biocidal Activity of the Nanoformulations?

**DOI:** 10.3390/pharmaceutics16010108

**Published:** 2024-01-14

**Authors:** Stefania Petralito, Stefania Garzoli, Elisa Ovidi, Valentina Laghezza Masci, Jordan Trilli, Barbara Bigi, Laura Di Muzio, Vito Cosimo Carriero, Maria Antonietta Casadei, Patrizia Paolicelli

**Affiliations:** 1 Department of Drug Chemistry and Technologies, Sapienza University of Rome, P.le Aldo Moro 5, 00185 Rome, Italy; stefania.petralito@uniroma1.it (S.P.); jordantrilli94@gmail.com (J.T.); barbara.bigi@uniroma1.it (B.B.); laura.dimuzio@uniroma1.it (L.D.M.); vitocosimo.carriero@uniroma1.it (V.C.C.); mariaantonietta.casadei@uniroma1.it (M.A.C.); patrizia.paolicelli@uniroma1.it (P.P.); 2 Department for the Innovation in Biological, Agrofood and Forestal Systems, Tuscia University, 01100 Viterbo, Italy; eovidi@unitus.it (E.O.); laghezzamasci@unitus.it (V.L.M.)

**Keywords:** stability, chemical composition, antibacterial activity, Ostwald ripening

## Abstract

In this work, *Lavandula x intermedia* essential oil (LEO) was encapsulated in lipid-based nanoemulsions (NanoLEO) using the solvent-displacement technique. In order to preserve the colloidal stability of the formulation, LEO was appropriately doped with the incorporation of different levels of a water-insoluble oil used as a ripening inhibitor. All the nanoemulsion samples were evaluated in terms of the impact of the water-insoluble oil on the nanoemulsion formation, physical–chemical properties, and antibacterial effectiveness against *E. coli* (Gram-negative) and *B. cereus* (Gram-positive). The presence of the inert oil added benefits to the formulations in terms of appearance, colloidal stability, and loss of volatile components. However, the antimicrobial activity of the nanoemulsions dramatically decreased with the ripening inhibitor addition, probably because it hampered the internalization of the antimicrobial components of LEO within the bacterial cell membranes, thus nullifying the delivery ability of the nanoemulsion formulation. On the contrary, the undoped NanoLEO formulation showed unaltered antibacterial activity in both *E. coli* and *B. cereus* up to 40 weeks from the preparation.

## 1. Introduction

In recent years, there has been a great interest in the design of novel eco-friendly essential oil-based antibacterial as alternatives to synthetic antimicrobial products [1,2]. Current excessive usage of traditional synthetic antimicrobial agents represents a potential threat to human health due to the risks related to the development of pathogen resistance mechanisms. EOs and their components have shown good effective potential against antimicrobial resistance [2,3,4]; in particular, a growing interest in the effective application of EOs in various fields, including therapy and environmental sanitization as natural biocide products, i.e., pesticides, disinfectants, and preservatives, has prompted researchers to focus several research studies to novel formulations with relevant potential for enhancing their chemical stability and water solubility [5,6,7]. In fact, for the most part, EO components, which derive from a great range of different chemical classes, are known to be highly volatile and susceptible to chemical degradation. Furthermore, the EO concentration required to attain effective antimicrobial activity can be about 100 times higher than that of standard synthetic antibiotics. For these reasons, their formulation requires strategies that are able to hinder the susceptibility of EOs to degradation while enhancing their effectiveness and providing a controlled release of their components [8,9,10]. 

Recently, many kinds of formulations containing natural and/or synthetic lipids have been designed and proposed to overcome the drawbacks of EOs. Among these formulations, nanoemulsions efficiently contribute to minimizing undesired modification of the EO components, protecting their integrity from high volatility and degradation reactions, which can reduce both their antimicrobial efficacy and hamper their applications [11,12,13]. Due to these facts, EO-in-water nanoemulsions could be considered an effective formulation strategy to fully exploit the antimicrobial potential of EOs. Every time a colloidal formulation is considered for a new treatment option, its capacity to preserve component composition unaltered over time, and its long-term colloidal stability has to be studied [14,15]. The colloidal properties of a nanoemulsion system, as well as its formation and stability, are strongly influenced by the physicochemical properties of the EO components. In particular, because of low viscosity, low interfacial tension, and high polarity, EOs tend to produce nanoemulsions with small droplet sizes [15]. However, at the same time, although such properties are desirable from the formulation point of view, nanoemulsions containing EOs usually present short-term stability due to destabilization phenomena, mainly Ostwald ripening (OR) or coalescence [16,17,18,19,20]. Different strategies have been reported for reducing the rate of OR of oil-in-water nanoemulsions, which represents the main aging process controlling their shelf life [21,22,23,24,25]. Numerous studies have shown that water-insoluble oils, such as medium-chain triglycerides (MCTs), can be successfully combined with EOs as “ripening inhibitors” in order to provide better colloidal stability to nanoemulsion-based delivery systems [7,26,27,28,29,30,31]. In fact, even though EOs are predominantly hydrophobic, they still possess a significant solubility in the aqueous phase. Therefore, by incorporating a second component of extremely low aqueous solubility in the oily phase, EO partitioning in the external aqueous phase and the consequent droplet growth due to OR can be dramatically reduced [21,32]. For these reasons, many authors mixed EOs and ripening-inhibitor oils to guarantee the long-term stability of the system. Although the addition of OR is a suitable strategy for nanoemulsion stability, some authors reported that their presence in the formulation can affect the efficiency of nanoemulsion formation, as well as the EO activity [22,29]. In order to bring light to this limitation, it is essential to understand the impact of the formulation on the formation, colloidal stability, and biological activity of EOs. Specifically, it is important to investigate whether and how the presence and the concentration of a ripening inhibitor may influence the formation and stability of a nanoemulsion, as well as the antimicrobial activity of the formulated EO. This information is of fundamental importance for the design and development of optimal and effective formulations. 

Recently, the solvent-displacement technique was adopted by our research group to formulate the *Lavandula x intermedia* EO (LEO) in the nanoemulsion (NanoLEO) [33]. Microbiological tests carried out to evaluate the antibacterial potential of NanoLEO revealed that LEO antimicrobial activity was enhanced both against Gram-positive (G^+^) and Gram-negative (G^−^) bacteria when formulated as a nanoemulsion [34,35]. Starting from these promising results, the objective of this study was to develop LEO-based nanoemulsions with low impact on the environment and human health for potential use in therapy or as a valid alternative to traditional biocidal products and investigate their stability over time, considering both the colloidal and biological perspective. To this end, LEO was doped with different levels of medium-chain triglycerides (MCTs) and used as a ripening inhibitor. The obtained nanoemulsions were tested to evaluate the impact of the formulation components on the formation, colloidal stability, chemical composition, and antimicrobial efficacy over nine months.

## 2. Materials and Methods

### 2.1. Materials

Soybean phosphatidylcholine (Phospholipon90; SPC) from Lipoid and Labrafac^TM^ lipophile WL 1349 (medium-chain triglycerides, MCT, and caprylic and capric acids) were kindly gifted by AVG and Gattefossé, respectively. 3-(4,5-dimethylthiazol-2-yl)-2,5-diphenyltetrazolium bromide (MTT), benzalkonium chloride, ethanol, acetone, and DMSO were obtained from Sigma-Aldrich (St. Louis, MO, USA). Pure commercial *Lavandula x intermedia* (lavandin) “Grosso” essential oil (LEO) was directly provided by Azienda Podere dell’Arco (Lazio Region, Italy).

### 2.2. Nanoemulsion Preparation

The nanoemulsions were prepared following a procedure reported in the literature and based on the solvent displacement technique [33]. Specifically, this method consists of the spontaneous emulsification of an organic phase (oil + surfactants) added to an aqueous phase maintained under gentle stirring at room temperature and without the aid of any external thermal or further mechanical energy source. In further detail, the organic phase consisted of 125 µL of LEO or mixtures of LEO and MCTs (LEO:MCT 3:1 *v:v* or LEO:MCT 1:3 *v:v*), 30 mg of SPC, and 4 mg of benzalkonium chloride dissolved in a solvent mixture composed of 9.5 mL of acetone and 0.5 mL of ethanol. The addition of the organic phase onto 10 mL of deionized water maintained under magnetic stirring led to the diffusion of the organic solvents, whereas surfactants moved to the water/oil interphase, thus leading to the immediate and spontaneous formation of the nanoemulsion, as evidenced by the milky appearance of the system. The organic solvents were removed by evaporation under reduced pressure until a final volume of 10 mL. Nanoemulsion prepared using LEO as an oily phase without the inert oil was the control formulation.

### 2.3. Physicochemical Characterization of Nanoemulsions

#### 2.3.1. Droplet Size and Size Distribution

Dynamic light scattering (DLS, Zetasizer Nano-ZS, Malvern Instruments, Worcester, UK) with a capability of measuring colloidal dispersions in the range of 0.3 nm–10 µm was used to determine the droplet size and polydispersity index (PDI) of the nanoemulsions. The DLS technique used a photon correlator spectrometer equipped with a 4 mW He/Ne laser source operating at λ = 633 nm. All measurements were performed at a scattering angle of 90° and were thermostatically controlled at 25 °C. The samples were opportunely diluted with demineralized water before analysis in order to avoid multiple scattering effects. Hydrodynamic diameter and polydispersity index values of the nanoemulsion formulations come from the mean of three different preparation batches ± standard deviation.

To assess the colloidal stability of the formulations, freshly prepared nanoemulsions were placed in dark-glass sealed vials and stored at 4 °C. At seven pre-established time points (0, 1, 2, 4, 6, 8, 10, and 40 weeks), the formulations were visually observed for phase separation or creaming, and, if any, size and size distribution were measured. 

#### 2.3.2. Determination of ζ-Potential

The droplet charge (ζ-potential) of the nanoemulsions was measured using particle microelectrophoresis (Zetasizer Nano ZS-90, Malvern Instruments, Worcester, UK). For zeta potential analysis, the samples were diluted with demineralized water prior to measurements in order to avoid multiple scattering effects.

#### 2.3.3. Optical Properties of Nanoemulsions

The opacity of nanoemulsions was expressed in terms of % of transmittance and determined by measurements of light attenuation over time in order to assess any potential change in the optical properties of the nanosystems. To this end, 1 mL of each formulation was diluted 1:100 using demineralized water prior to the analysis. The percent transmittance was measured using spectrophotometry UV/Vis at λ = 633 nm with a Lambda 40 Perkin Elmer (Waltham, MA, USA), spectrophotometer. The measurement was repeated for each formulation in triplicate, and their average values ± SD were calculated.

### 2.4. Chemical Analyses

To investigate the volatile composition of encapsulated LEO over time, a Perkin-Elmer (Waltham, MA, USA) Headspace Turbomatrix 40 autosampler connected to a Clarus 500 GC-MS was used. For the separation, a Varian Factor Four VF-1 (60 m × 0.32 mm, 1.0 μm of film thickness) capillary column was used. The GC temperature program was as follows: 40 °C to 220 °C at a rate of 5 °C/min, held for 10 min. Helium was used as carrier gas at a flow rate of 1 mL/min. The mass spectra were recorded at 70 eV (EI) and were scanned in the range of 40–450 *m/z*. The ion source and the connection parts temperature was 220 °C. The headspace procedure was performed following Garzoli et al. with slight modifications [33]. The peak areas of the FID signal were used to calculate the relative percentages of the components without the use of an internal standard or any factor correction. The identification of the components was performed by comparing the mass spectra for each compound with those reported on the MS library search (Wiley and Nist 11). Furthermore, linear retention indices (LRIs) of each compound were calculated using a mixture of n-alkanes (C_8_–C_30_, Ultrasci) at the same operating conditions reported above. All analyses were repeated twice.

### 2.5. Bacteria Strains and Culture Conditions

*E. coli* ATCC 25922 (Gram-negative, G^−^) and *B. cereus* ATCC 10876 (Gram-positive, G^+^) were used to assess the antimicrobial activity. Bacteria were grown in Lysogeny Agar at the growth temperature (37 °C and 26 °C for *E. coli* and *B. cereus*, respectively).

### 2.6. Determination of Antibacterial Activity

The microdilution test was used to determine the antibacterial activity of encapsulated LEO over time against *E. coli* and *B. cereus*. Prior to testing, bacteria were grown on Lysogeny Agar at the appropriate temperature, and after 24 h, they were collected and used to prepare the inoculum (10^6^ CFU/mL). For each bacterial strain, the lowest concentration of NanoLEO sample that inhibited the bacterial growth (Minimum Inhibitory Concentration, MIC) [33] was defined for each of the seven-time points (T0, T1, T2, T3, T4, T5, and T6, corresponding to 0, 1, 2, 4, 6, 10, and 40 weeks of storage at 4 °C). 

The 96-well microplates were seeded with 50 µL of the inoculum, and twelve concentrations of the sample and relative controls (a no-treatment control, a positive control with twelve two-fold dilutions of gentamicin from 2 mM to 2 × 10^−3^ µM, and a sterility control without bacteria) were added under appropriate culture conditions. All tests were repeated in triplicate. Bacterial growth in each well was determined by 3-(4,5-dimethylthiazol-2-yl)-2,5-diphenyltetrazolium bromide (MTT).

In order to define the Minimum Bactericidal Concentration, which corresponds to the minimum concentration of encapsulated essential oil that kills bacteria cells, 10 µL of each of four dilutions of broth without bacterial growth were re-cultured in an agarized petri dish for 24 h at the corresponding growth temperature. At each considered time point, the detected MBC was recorded [33].

### 2.7. Statistical Analysis

Data concerning the physical–chemical properties of nanoemulsions were statistically analyzed using Student’s *t*-test. The GC/MS results were expressed as means ± standard deviation (SD), and the Anova test (One-way analysis of variance test) followed by Tukey’s HSD test was used to analyze significant differences among means (*p* < 0.01; * *p* < 0.05).

## 3. Results and Discussion

### 3.1. Nanoemulsion Properties

Droplet size and size distribution are usually expressed as the mean particle diameter and polydispersity index (PDI) that describes the width or spread of the particle size distribution. These features impact the physicochemical properties of the nanoemulsion, such as its appearance, texture, and shelf-life [36]. In particular, based on the mean particle diameter, nanoemulsions could appreciably differ in their optical properties, such as the turbidity attribute. Furthermore, the nature and composition of the droplets, the concentration of the oily components, their density, and also the interfacial characteristics could lead to substantial differences in the optical properties of nanoemulsions. For these reasons, nanoemulsions can appear optically transparent or weakly turbid [37], and any loss of components from the droplets over time or changes in the thickness of the stabilizing interfacial film formed by amphiphilic molecules could bring variation in the droplet size and, as a consequence, could impact the optical properties in addition to the physical stability of these colloidal systems. 

Under the adopted experimental conditions, the solvent displacement technique allowed for the emulsification of LEO to occur spontaneously in a few minutes, leading to an opaque/milky white o/w nanoemulsion characterized by nanodroplets having a mean size of 323 ± 3 nm and a PDI of 0.103 ± 0.016 (Table 1), compatible with the milky appearance of NanoLEO formulation. Nanoemulsions can be optically clear or milky, depending on the size of the droplets. As a matter of fact, the scattering intensity scales with the droplet size and the number of multiple scattering scales with the number of interfaces are related to the oil volume fraction. All these effects prevent the transmission of light through the emulsion, determining the optical property of the obtained nanoemulsions [36].

The main factors that influence the droplet size and the properties of nanoemulsions are the amount of both energy applied and surfactants used. In particular, high-energy emulsification methods allow for the emulsification of the oil phase in smaller droplets. However, the high-energy approach dissipates a substantial quantity of energy as friction losses due to the high shear rates; this energy is usually transformed to heat, which may increase the emulsion temperature significantly and, consequently, may affect the stability of the oil components [15,33]. In the same way, an increase in the surfactant-to-oil ratio generally results in a decrease in droplet size, but large amounts of surfactants may raise some safety issues.

Furthermore, although it is true that larger surface area and smaller droplets (<200 nm) may be beneficial for the biological effects of the nanoemulsions, it is important to consider that smaller droplets may promote the Ostwald ripening phenomena in which smaller particles dissolve and deposit on larger particles [38]. In addition, the milky appearance of the obtained LEO nanoemulsion could represent, in our opinion, a more desirable property with respect to the optical transparency obtained with other experimental techniques or other oil-to-water volume ratio or other surfactant-to-oil ratio, as it may offer a better protection to the LEO components against the exposure to environmental factors, including UV/Vis radiation [33,36]. Indeed, the natural sunlight spectrum is a potential enhancer of the decomposition of pharmaceuticals, leading to potentially toxic products. Although such effects could be avoided using opaque vials for storage, this alternative approach hampers visual inspection of the final product. For all these reasons, in this work, a low-energy method was adopted to prepare milky nanoemulsions.

An inert oil composed of MCTs was added in the oil phase, partially replacing LEO volume, in order to maintain a constant water-to-oil ratio in the formulation. Maintaining the same surfactant and co-surfactant concentration in the formulation, the spontaneous and rapid formation of nanoemulsions was obtained independently from the level of the ripening inhibitor added to LEO. This means that SPC and benzalkonium [39,40] were still able to move to the oil/water interphase, leading to the immediate and spontaneous formation of the nanoemulsion, although droplet size and size distribution resulted dependent on the LEO:MCT ratio (Table 1). 

In particular, using LEO:MCT at a ratio of 3:1 and 1:3, the obtained samples were 324 ± 2 nm or 244 ± 3 nm in size, with a PDI of 0.100 ± 0.017 or 0.112 ± 0.010, respectively, indicating that droplet size was inversely correlated with the concentration of OR inhibitor, however, still compatible with the formation of a milky nanoemulsion.

The decrease in mean particle diameter with increasing MCT level (LEO:MCT at a ratio of 1:3 *v:v*) can be attributed to the fact that the presence of the MCT facilitated the formation of small droplets during spontaneous emulsification [26].

Zeta potential refers to the droplet charge, which is indicative of the degree of repulsion between particles, and it contributes to the colloidal stability of nanoemulsion. All tested samples showed a high positive value of zeta potential, probably due to the cationic group of the co-surfactant component (benzalkonium chloride), and progressively increased with the amount of MCT present in the formulation ranging from +38.0 mV of the control NanoLEO without OR inhibitor, to +39.6 mV and +44.8 mV, when the concentration of the inert oil gradually increased. 

These results indicate that LEO, MCTs, SPC, benzalkonium chloride, and the different concentrations used in the aqueous phase are able to form, under the adopted experimental conditions, stable nanoemulsions with good stability against destabilizing mechanisms, including OR.

**Figure 1:**
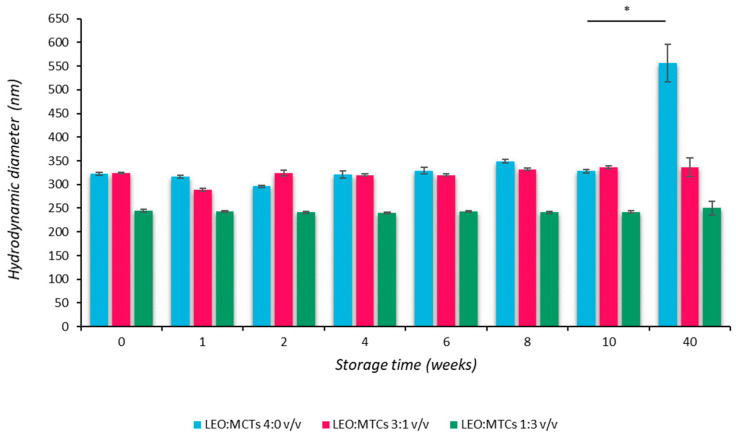
Influence of MCT on the mean particle diameter of LEO-in-water nanoemulsions at different LEO:MCT ratios. Nanoemulsions were stored at 4 °C for up to 40 weeks and regularly analyzed for particle size. More specifically, mean hydrodynamic diameter was measured at the beginning of this study (time zero) and after different weeks of storage at 4 °C. * *p* < 0.05.

In fact, the ripening effect is negligible, as modest changes in droplet size distribution were observed over time. In particular, after storage at 4 °C for 10 weeks, the overall appearance of the nanoemulsions remained fairly similar to that of the freshly prepared systems. All nanoemulsions did not change appreciably during storage, and there was no visible evidence of droplet growing, as demonstrated by the mean hydrodynamic droplet diameters that remained closely similar to those of the freshly prepared systems (Figure 1). After 40 weeks, the control NanoLEO exhibited an increase in the mean particle diameter (Z-average), and the corresponding size distributions showed a progressive broadening. According to this behavior, we speculate that the smaller droplets, which have larger solubility when compared with the larger ones with time, diffuse to the continuous phase, and their molecules become deposited on the larger droplets. After 40 weeks, the droplet size distribution of the control NanoLEO shifts to larger values. More specifically, the rise in the average droplet size can be attributed to the Ostwald ripening process. During this process, drop size distributions undergo general broadening since some of the drops become smaller while others grow.

The susceptibility of a nanoemulsion to this instability mechanism can be attributed to the solubility of the smaller oil droplets due to the curvature effect and to the appreciable water solubility of some components of the EO in the aqueous phase. The major constituents of LEO have appreciable water solubility, and over time, they could promote the movement of these components from smaller to larger droplets through the aqueous phase [32,33], irrespective of the zeta potential values.

The inclusion of MCTs in the oil droplets (regardless of the different volume ratios) slowed down the droplet growth, and consequently, the average droplet size remained unaltered also after 40 weeks of storage. 

In the absence of MCTs, the Otswald ripening phenomenon could be accompanied by a simultaneous loss of the volatile oil fraction over time with a consequent decrease in the dispersed phase concentration [29,32]. 

**Figure 2:**
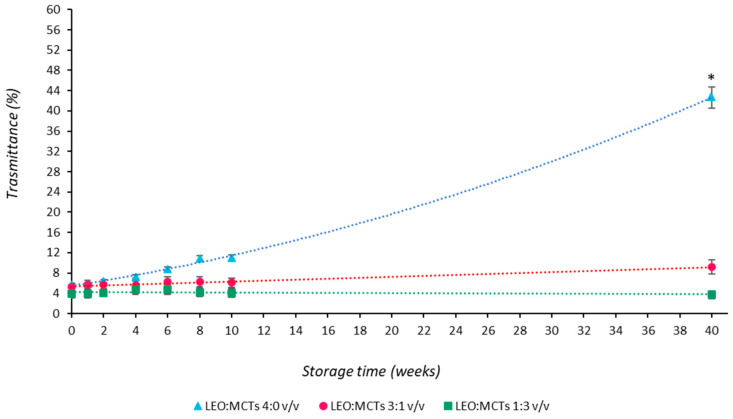
Influence of MCTs on percentage of transmittance of LEO-in-water nanoemulsions at different LEO:MCT ratios. Nanoemulsions were stored at 4 °C for up to 40 weeks and regularly analyzed for optical properties. * *p* < 0.05.

The loss of the volatile oil fraction might have many undesirable effects on the potential of the nanoemulsionated systems, including an undesired reduction in the antimicrobial activity of the formulation. Furthermore, if the milky-colored nanoemulsions lose part of their dispersed oil droplets, they can become optically clear and so more susceptible to environmental conditions [40]. In order to control the loss of the EO components from the formulations over time, the optical properties of the nanoemulsions have been monitored over time since many authors assume that they exhibit an interesting volume fraction distribution dependence [36]. In light of this, transmittance percentages were measured to follow the evolution of the volume fraction distribution in the colloidal samples. Results reported in Figure 2 show a significant modification of the optical properties only for the control formulation (NanoLEO), which became less milky over time. 

In particular, all freshly prepared nanoemulsions showed a percentage of transmittance of around 5%, but only the control NanoLEO formulation changed from a milky appearance toward a clearer optical aspect, as demonstrated by the sharp increase in % of transmittance after 40 weeks. This result confirms that the reduction in the volume fraction, due to the loss of the volatile oil components over time, can be considered the additional factor influencing the emulsion optical properties besides the droplet size. In order to further support these considerations, headspace gas chromatography–mass spectrometry was used as a powerful and efficient way to profile the composition of complex, volatile components over time. 

### 3.2. HS-GC/MS Analyses

As evident by HS-GC/MS analyses, twenty-three compounds were identified and listed in Table 2. Monoterpenes oxygenated were the most abundant, ranging from 87.3 to 94.0%, followed by monoterpene hydrocarbons whose percentage values ranged from 5.4 to 10.5%. Sesquiterpenes compounds, present in small percentages, began to appear at time T2 (0.2%), reaching their highest value at time T6 (2.0%). Linalol, camphor, and 1,8-cineole were the principal oxygenated monoterpenes whose percentage trend over time was not the same; in fact, linalool and 1,8-cineole increased from T0 (53.3% and 7.6%) to T6 (57.6% and 11.4%), respectively, while camphor decreased from T6 (8.2%) to T0 (21.1%) and measured its highest value at T1 (22.7%). Among the monoterpene hydrocarbons, β-myrcene was the most representative, and it, too, showed an increasing trend over time (T0: 3.5%; T6: 5.6%).

From a qualitative point of view, the number of detected compounds increased from T2, reaching the highest number of identified terpenoids, such as α-phellandrene, lavandulyl acetate, nerol acetate, (-)-β-bourbonene, and cis-β-farnesene at T6.

Moreover, for the two most abundant compounds, such as linalool and 1,8-cineole [38,41,42], a progressive increase in their percentage in the airborne phase is observed, which translates, over time, into a gradual loss of these components from the formulation. This phenomenon is closely related to their degree of solubility in water. In fact, on the other side, camphor, a compound insoluble in water and linalyl acetate much less soluble than linalool or 1,8-cineole follows an opposite trend characterized by a decreasing relative concentration over time.

### 3.3. Nanoemulsion Biocidal Activity

In a previous study, NanoLEO preparation proved to be very active when tested on *E. coli* and *B. cereus*. A marked increase in antimicrobial activity, in fact, was observed on both G^+^ (MIC value of 0.01% against *B. cereus*) and G^−^ (MIC value of 0.037% against *E. coli*) bacteria for LEO. The increase in the antimicrobial activity could be attributed to the increased specific surface area due to the reduced droplet size. However, the nanoemulsions containing LEO:MCT 4:0 *v/v* and LEO:MCT 3:1 *v/v* both had similar diameters but very different antimicrobial activities, which does not support this hypothesis. This result could be explained, considering that the composition of the oil phase also had a crucial impact on the antimicrobial activity of the nanoemulsions [38,43,44], as determined by measuring their MICs, which resulted in 0.078% compared to *B. cereus* and higher than 0.313% compared to *E. coli*, respectively (Table 3). MCTs used in the oil phase of the nanoemulsions dramatically affected the antimicrobial activity of the nanosystem: indeed, as the MCTs level increased, there was an increase in the MIC values, indicating that the antimicrobial efficacy of the tested samples was decreased. 

The observed effect could be attributed to the impact of the ripening inhibitor on the partitioning of the antimicrobial components of LEO between the nanoemulsion droplets and the bacterial cell membranes [32]. In particular, the ineffectiveness was attributed to the inability to deliver sufficient levels of hydrophobic antimicrobial LEO from the oil droplets into the microbial cell membranes when the oil droplets interact with the surfaces of the microbial cell [32]. Indeed, MCTs-free and MCTs-doped nanoemulsions were characterized by similar zeta potential values; therefore, similar electrostatic interactions could be expected among microbial cell membranes and the different nanoemulsion droplets. Consequently, the different activity might be attributed to differences in the release rate of LEO active components from the formulations. It is likely that the active components remained entrapped within the oil droplets, and only ineffective concentrations were released from the MCT-doped formulations. Instead, the MCT-free formulation (LEO:MCT 4:0 *v/v*) showed activity on bacterial vitality. Indeed, no variation in the MIC values of NanoLEO was observed over time despite the partial loss of volatile components and the alteration of the droplet size distribution. This observation underlines the importance of preserving the mechanisms involved in the mass transport of biocidal compounds from essential oil nanoemulsions to bacterial cell membranes while avoiding the loss of volatile components from the formulation because both these factors can cause a significant decrease in biocidal activity.

## 4. Conclusions

In this study, the impact of ripening inhibitors on the formation, stability, and antimicrobial activity of NanoLEO, formulated by spontaneous emulsification, was investigated. The presence of the inert oil added benefits to the formulations in terms of appearance, stability, and loss of volatile components. However, when different proportions of MCTs were added to colloidal formulations, the MIC increased, suggesting that the LEO nanoemulsions became less effective at inhibiting bacterial growth. The decrease in MIC value when the MCT was added could be due to changes in the equilibrium partitioning of the more polar antimicrobial compounds between the oil droplets of the nanoemulsions and the bacterial cell membranes.

For this reason, measuring the impact of carrier oil composition on the partitioning behavior of the antimicrobial compounds in the nanoemulsions, as well as the knowledge of the antimicrobial delivery mechanism, became of paramount importance in the design of effective antimicrobial EO-based nanoemulsions.

## Figures and Tables

**Table 1:** Effect of MCTs content on the hydrodynamic mean diameter, polydispersity index (PDI), and zeta potential values of LEO-based nanoemulsions measured soon after preparation (time zero, T0).

SAMPLE	LEO:MCTs Volume Ratio (*v/v*)	Hydrodynamic Diameter (nm)	PDI	Zeta Potential (mV)
Sample 1	4:0	323 ± 3	0.103 ± 0.016	+38.1 ± 0.2
Sample 2	3:1	324 ± 2	0.100 ± 0.017	+39.6 ± 3.0
Sample 3	1:3	244 ± 3	0.112 ± 0.010	+44.8 ± 0.8

**Table 2:** Vapor phase chemical composition (percentage mean values ± SD) of NanoLEO.

COMPONENT ^1^	LRI ^2^	LRI ^3^	T0%	T1%	T2%	T3%	T4%	T5%	T6%
1-octen-3-ol	962	964	-	-	0.2 ^a^ ± 0.02	0.1 ^b^ ± 0.00	0.1 ^b^ ± 0.01	-	-
β-myrcene	971	978	3.5 ^a^ ± 0.03	2.8 ^b^ ± 0.04	3.8 ^c^ ± 0.03	3.5 ^a^ ± 0.03	3.3 ^d^ ± 0.04	4.0 ^e^ ± 0.02	5.6 ^f^ ± 0.03
acetic acid, hexyl ester	1000	1002	-	-	0.3 ^a^ ± 0.02	tr	0.1 ^bc^ ± 0.02	0.1 ^c^ ± 0.00	0.2 ^d^ ± 0.01
α-phellandrene	1003	1005	-	-	tr	tr	tr	-	0.1 ± 0.00
α-terpinene	1021	1019	-	-	tr	tr	tr	-	-
p-cymene	1019	1023	-	-	0.1 ^a^ ± 0.00	0.1 ^a^ ± 0.00	0.1 ^a^ ± 0.00	0.2 ^bc^ ± 0.01	0.2 ^c^ ± 0.01
1,8-cineole	1027	1025	7.6 ^a^ ± 0.05	9.9 ^b^ ± 0.06	8.4 ^c^ ± 0.04	9.5 ^d^ ± 0.05	9.2 ^e^ ± 0.05	10.8 ^f^ ± 0.07	11.4 ^g^ ± 0.08
cis-β-ocimene	1032	1032	2.2 ^a^ ± 0.03	1.7 ^b^ ± 0.02	2.1 ^a^ ± 0.03	2.2 ^a^ ± 0.04	2.1 ^a^ ± 0.04	2.5 ^c^ ± 0.03	2.8 ^d^ ± 0.05
trans-β-ocimene	1048	1043	1.5 ^a^ ± 0.04	0.9 ^b^ ± 0.03	1.2 ^cde^ ± 0.04	1.3 ^de^ ± 0.03	1.2 ^e^ ± 0.04	1.5 ^a^ ± 0.02	1.8 ^f^ ± 0.05
trans-linalol oxide	1061	1056	0.7 ^a^ ± 0.02	0.7 ^a^ ± 0.02	0.9 ^a^ ± 0.03	0.7 ^a^ ± 0.02	0.8 ^a^ ± 0.02	1.0 ^a^ ± 0.03	1.1 ^a^ ± 0.04
cis-linalol oxide	1070	1066	1.2 ^a^ ± 0.02	1.2 ^a^ ± 0.03	1.3 ^b^ ± 0.04	1.0 ^c^ ± 002	1.2 ^a^ ± 0.02	1.7 ^d^ ± 0.03	1.9 ^e^ ± 0.04
Linalol	1099	1092	53.3 ^a^ ± 5.90	52.0 ^bca^ ± 6.01	51.5 ^ca^ ± 5.62	52.5 ^a^ ± 5.74	54.0 ^ad^ ± 6.01	55.2 ^d^ ± 5.81	57.6 ^e^ ± 6.52
propanoic acid, 2-methyl-, hexyl ester	1134	1132	-	-	0.2 ^a^ ± 0.01	0.1 ^b^ ± 0.01	-	-	-
Camphor	1141	1139	21.1 ^a^ ± 0.09	22.7 ^b^ ± 0.10	9.1 ^ce^ ± 0.07	9.8 ^de^ ± 0.08	9.9 ^e^ ± 0.06	9.0 ^f^ ± 0.06	8.2 ^g^ ± 0.05
Lavandulol	1152	1148	2.4 ^a^* ± 0.03	2.3 ^b^ ± 0.03	0.3 ^cg^* ± 0.02	0.2 ^def^* ± 0.01	0.2 ^ef^* ± 0.01	0.2 ^f^ ± 0.02	0.3 ^g^ ± 0.02
terpinen-4-ol	1173	1168	-	-	2.7 ^a^ ± 0.03	3.0 ^bcd^ ± 0.02	3.1 ^cd^ ± 0.04	3.0 ^d^ ± 0.02	2.7 ^a^ ± 0.04
Borneol	1175	1169	1.1 ^a^ ± 0.01	1.1 ^a^ ± 0.01	0.2 ^b^ ± 0.01	2.3 ^ce^ ± 0.02	2.4 ^d^ ± 0.02	2.2 ^e^ ± 0.01	1.1 ^a^ ± 0.02
α-terpineol	1186	1183	2.7 ^a^ ± 0.03	2.7 ^a^ ± 0.04	0.9 ^bcd^ ± 0.04	0.9 ^cd^ ± 0.03	0.9 ^d^ ± 0.03	0.5 ^e^ ± 0.02	0.1 ^f^ ± 0.00
linalyl acetate	1256	1252	2.1 ^a^ ± 0.02	1.4 ^b^ ± 0.03	10.9 ^c^ ± 0.07	8.4 ^d^ ± 0.05	7.2 ^e^ ± 0.04	2.5 ^f^ ± 0.03	0.2 ^g^ ± 0.02
lavandulyl acetate	1269	1271	-	-	2.8 ^a^ ± 0.02	2.2 ^b^ ± 0.03	2.0 ^cd^ ± 0.02	1.9 ^de^ ± 0.02	1.8 ^e^ ± 0.03
nerol acetate	1364	1363	-	-	2.6 ^a^ ± 0.05	2.0 ^bc^ ± 0.03	1.9 ^cd^ ± 0.03	1.8 ^d^ ± 0.03	0.9 ^e^ ± 0.02
(-)-β-bourbonene	1392	1390	-	-	-	tr	tr	1.5 ^a^ ± 0.03	1.9 ^b^ ± 0.04
cis-β-farnesene	1455	1451	-	-	0.3 ^a^ ± 0.02	0.2 ^b^ ± 0.02	0.2 ^c^ ± 0.02	0.1 ^de^ ± 0.00	0.1 ^e^ ± 0.00
SUM			99.4	99.4	99.8	100.0	99.9	99.7	100.0
Monoterpene hydrocarbons			7.2	5.4	7.2	7.1	6.7	8.2	10.5
Monoterpenes oxygenated			92.2	94.0	91.6	92.5	92.8	89.8	87.3
Sesquiterpene hydrocarbons			-	-	0.3	0.2	0.2	1.6	2.0
Other			-	-	0.7	0.2	0.2	0.1	0.2

^1^ The components are reported according to their elution order on apolar column; ^2^ Linear Retention Indices measured on apolar column; ^3^ Linear Retention indices from the literature; -: Not detected; tr: Percentage mean values < 0.1%. Data are means ± standard deviation of three (n = 3) replicates. Means with different letters in the same column are significantly different (Student’s *t*-test, *p* < 0.01; * *p* < 0.05).

**Table 3:** MIC and MBC of Nano LEO. Values are expressed as the percentage (%) of LEO in the nanoformulation.

	Time Points	T0	T1	T2	T3	T4	T5	T6
*B. cereus*	MIC (%)	0.078	0.078	0.078	0.078	0.078	0.078	0.078
	MBC (%)	0.313	0.313	0.313	0.313	0.313	0.313	0.313
*E. coli*	MIC (%)	>0.313	>0.313	>0.313	>0.313	>0.313	>0.313	>0.313
	MBC (%)	-	-	-	-	-	-	-

MIC: Minimum Inhibitory Concentration; MBC: Minimum Bactericidal Concentration; -: not executed. MIC and MBC values for gentamicin used as positive control were 0.78 × 10^−3^ mM for *B. cereus* and 6.7 × 10^−3^ mM for *E. coli* for each time point considered.

## Data Availability

All generated data are included in this article.
